# Efficacy of Standard Biophysical Tests in Assessing Fetal Oxygenation Status

**DOI:** 10.7759/cureus.96591

**Published:** 2025-11-11

**Authors:** Branka Cancarevic Djajic, Dragica Draganovic, Tanja Milic-Radic, Suzana Sobot, Bojana Popovic, Jelena Milidragovic, Milan Vjestica, Stefan Bozic, Ljubisa Preradovic

**Affiliations:** 1 Obstetrics and Gynecology, Faculty of Medicine, University of Banja Luka, Banja Luka, BIH; 2 Obstetrics and Gynecology, University Clinical Center of the Republic of Srpska, Banja Luka, BIH; 3 Anesthesiology and Critical Care, University Clinical Center of the Republic of Srpska, Banja Luka, BIH; 4 Computer Sciences, Faculty of Architecture, Civil Engineering and Geodesy, University of Banja Luka, Banja Luka, BIH

**Keywords:** acidosis, apgar score, cardiotocography, fetal biophysical profile, perinatal outcome, umbilical artery blood ph

## Abstract

Introduction: Timely recognition of fetal oxygenation disorders is essential to prevent irreversible injury. Cardiotocography (CTG) and biophysical profile (BPP) are widely used for fetal surveillance, yet their diagnostic and predictive performance relative to umbilical artery (UA) pH and neonatal outcomes warrants further evaluation. This study assessed the efficacy of CTG and BPP in predicting fetal acidosis and a low one-minute Apgar score (AS) and examined their value for identifying fetal hypoxia and asphyxia.

Material and methods: We conducted a retrospective study of 119 hospitalized pregnant women at the Department of Perinatology, University Clinical Center of the Republic of Srpska in Banja Luka, with gestational age ≥ 27 weeks, singleton pregnancies, and no fetal malformations. CTG tracings were classified as normal, equivocal, or pathological. The BPP was scored (0-10) and categorized as normal, equivocal, or pathological. We correlated test results with UA pH (acidosis < 7.20; pre-acidosis 7.20-7.24; normal ≥ 7.25) and the one-minute AS. We calculated sensitivity, specificity, positive predictive value, negative predictive value, and accuracy, and used appropriate significance tests.

Results: CTG and BPP showed similar predictive performance for detecting acidosis and a low one-minute AS. Both methods demonstrated statistically significant associations with UA pH and AS (p < 0.001). Perinatal mortality was 1.68% and morbidity was 24.37%; fatal outcomes were associated with pathological findings on both tests, acidosis, and a low AS.

Conclusion: CTG and BPP are useful predictors of fetal hypoxia and asphyxia. Integrated use and careful interpretation of both tests can aid in identifying compromised fetuses and guiding timely decisions about the mode and timing of delivery, with the potential to reduce adverse perinatal outcomes.

## Introduction

The perinatal period begins at conception and includes pregnancy, delivery, and the first seven days after delivery. According to the World Health Organization, the perinatal period spans from 22 completed weeks of gestation and ends seven completed days after birth [1]. It is a time of significant vulnerability. The primary objective of modern perinatal care is to achieve the birth of a viable child with normal psychomotor development while safeguarding maternal health [2]. Because hypoxia is a leading cause of perinatal morbidity (defined as a neonatal intensive care unit stay for more than 24 hours) and mortality (defined as fetal death and early neonatal death in the first seven days of life), contributing to 50-60% of cases, assessment of fetal oxygenation is central to perinatal surveillance [2]. Timely recognition of oxygenation abnormalities is essential to guide delivery before irreversible fetal injury occurs. A major goal of antenatal assessment is the detection and prevention of hypoxic-ischemic injury and fetal demise [2].

Maintaining adequate fetal oxygenation is necessary for normal metabolism and development. Under hypoxic conditions, anaerobic glycolysis generates lactate and leads to metabolic acidosis [2-4]. The most accurate indicators of neonatal acid-base status are umbilical artery (UA) pH, base excess, and standard bicarbonate. UA pH, in particular, is considered the gold standard for objective assessment of fetal hypoxia at delivery [4].

In clinical practice, non-invasive monitoring methods such as cardiotocography (CTG) and the biophysical profile (BPP) help identify at-risk fetuses. CTG enables continuous evaluation of fetal heart rate and reactivity, whereas the BPP integrates ultrasound parameters with CTG to assess overall fetal well-being [5].

Despite their widespread use, the ability of CTG and BPP to predict the actual fetal acid-base state varies. The literature increasingly emphasizes validating these tools by correlating their findings with UA pH and the one-minute Apgar score (AS) [5,6]. Although simple and rapid, the AS can indirectly indicate prior intrauterine hypoxia at one minute, particularly when the score is three or lower and persists for more than five minutes, which aligns with criteria from the American College of Obstetricians and Gynecologists (ACOG) for perinatal hypoxia [3,5]. The definition of perinatal hypoxia of the newborn today includes the following criteria: severe umbilical arterial metabolic or mixed acidosis (pH less than 7.0), persistent AS of 0 to 3 lasting longer than five minutes, neonatal neurological sequelae (seizures, hypotonia, coma) or hypoxic-ischemic encephalopathy, and proven multiorgan dysfunction in the early neonatal period (cardiovascular system, gastrointestinal tract, respiratory and hematopoietic system) [3,5]. For clinical use, fetal asphyxia is considered a condition of impaired gas exchange that leads to progressive hypoxemia and hypercapnia with significant metabolic acidosis [7].

The objective of this study was to evaluate the diagnostic performance of CTG and BPP in assessing fetal oxygenation by correlating their findings with UA pH values and the one-minute AS. We also aimed to determine their diagnostic and predictive value for identifying fetal hypoxia and asphyxia.

## Materials and methods

This retrospective study enrolled 119 pregnant women hospitalized at the Department of Perinatology, Clinic of Obstetrics and Gynecology, University Clinical Center of the Republic of Srpska in Banja Luka from January 1, 2025, to July 1, 2025. Secondary data were used, collected from the medical records. The institutional review board ethics committee approved the study (approval no. 01-19-365-2/25, date of approval: August 11, 2025). Participants varied in age and parity and met the following inclusion criteria: reliable gestational age ≥ 27 weeks at delivery, singleton pregnancy without congenital fetal malformations, and a range of clinical diagnoses. 

We assessed fetal oxygenation using non-invasive clinical monitoring (BPP, CTG, AS) and biochemical markers (acid-base state of the UA blood). Fetal well-being was monitored with CTG and the BPP. We evaluated intrapartum fetal status using UA blood pH and the one-minute AS to determine the diagnostic efficacy of these methods.

CTG

We used CTG as a standard method to monitor fetal heart activity during pregnancy and labor. Cardiotocographic analysis (non-stress test (NST)) was performed with a Hewlett-Packard 8040A device (Hewlett-Packard, Bothell, USA) at a paper speed of 1 cm/min for a standard duration of 30 minutes, or for 15 to 20 minutes if the tracing was normal. We analyzed baseline fetal heart rate, variability, presence of accelerations, and early, late, and variable decelerations. CTG tracings were classified as reactive (normal) or non-reactive (encompassing equivocal and pathological findings). We further evaluated tracings with the Kuvačić-Dražančić CTG index [8] and categorized them as normal (0-2 points), equivocal (3-4 points), or pathological (≥ 5 points). We defined a pathological CTG as the presence of persistent tachycardia, reduced variability, and late decelerations.

BPP

We determined the BPP by combining ultrasound and CTG evaluation according to Manning, assessing five components over 30 minutes: fetal breathing movements, gross body movements, fetal tone, amniotic fluid volume, and the NST result [9]. Each component was scored 0 (pathological) or 2 (normal), with a maximum score of 10. We classified the total as normal (8 to 10 points), equivocal (4 to 6 points), or pathological (0 to 2 points). A BPP score less than or equal to 6 indicated potential fetal decompensation and an elevated risk of acidosis [6].

UA blood pH

We collected UA blood samples immediately after delivery. Prior to the newborn’s first breath, a segment of the umbilical cord measuring 20 to 25 cm was double-clamped, and blood was collected from the UA into heparinized tubes (Type D 551/12.5/200; Radiometer, Copenhagen, Denmark). The acid-base status of the neonate was analyzed using an ABL2 blood gas analyzer (Radiometer, Copenhagen, Denmark). We analyzed acid-base parameters, including pH, base excess, and standard bicarbonate. We categorized UA pH as normal (pH ≥ 7.25), pre-acidosis (pH 7.20-7.24), or acidosis (pH < 7.20) [10,11].

One-minute AS

We assessed neonatal vitality at one minute after delivery using the standard AS scale (heart rate, respiratory effort, muscle tone, reflex irritability, and skin color). We classified one-minute AS as 8-10 (good vitality), 4-7 (mild depression/asphyxia),1-3 (severe depression/asphyxia), and 0 (apparent stillbirth) [12,13]. There were no apparent stillbirths in this study.

We included biophysical tests performed on the day of delivery; when multiple tests were available, we used the final assessment before delivery. We then calculated the predictive value of CTG and BPP for fetal oxygenation status.

Statistical analysis

The data were analyzed with descriptive statistics and appropriate inferential tests using IBM SPSS Statistics for Windows, Version 22.0 (IBM Corp., Armonk, USA). We assessed associations between categorical variables with the chi-square test of independence. For 2×2 contingency tables, we applied Yates’ correction for continuity. When any expected cell frequency in a 2×2 table was less than five, we used Fisher’s exact test. All tests were two-sided, and p < 0.05 was considered statistically significant.

Diagnostic performance was evaluated by calculating sensitivity (Se), specificity (Sp), positive predictive value (PPV), negative predictive value (NPV), and overall accuracy. We also determined false-positive (FP) and false-negative (FN) rates. We derived these parameters from Table 1.

**Table 1:** Assistance table for calculation of SE, SP, PPV, NPV and accuracy SE, sensitivity; SP, specificity; PPV, positive predictive value; NPV, negative predictive value; TP, true positive; FP, false positive; FN, false negative; TN, true negative

Test result	Condition present	Condition absent
Normal	TP (a)	FP (b)
Abnormal	FN (c)	TN (d)

The formulas were as follows:

$\text{Se} = \frac{a}{a + c}, \quad\text{Sp} = \frac{d}{b + d}, \quad\text{PPV} = \frac{a}{a + b}, \quad\text{NPV} = \frac{d}{c + d}, \quad\text{Accuracy} = \frac{a + d}{a + b + c + d}$

## Results

We observed normal CTG findings in 84 (70.6%) participants; within this group, 58 (69.0%) neonates had a normal UA pH. Among 12 (10.1%) participants with an equivocal CTG, UA pH values were evenly distributed across the normal, pre-acidosis, and acidosis categories. In the subgroup with pathological CTG findings, eight (34.8%) neonates had acidosis, while eight (34.8%) had a normal UA pH (Table 2). A chi-square test with Yates’ correction for continuity showed a statistically significant association between the CTG category and UA pH (χ² = 9.136, p = 0.003).

**Table 2:** Cardiotocographic finding and UA blood pH *p-value and χ^2^ value obtained using the chi-square test with Yates’ correction for continuity, with p < 0.05 considered significant. CTG, cardiotocography; UA, umbilical artery

CTG	pH UA			Total, n (%)	χ^2^	p-value*
	Acidosis, n (%)	Pre-acidosis, n (%)	Normal, n (%)			
Normal	8 (9.5%)	18 (21.4%)	58 (69.0%)	84 (70.6%)	9.136	0.003
Equivocal	4 (33.3%)	4 (33.3%)	4 (33.3%)	12 (10.1%)		
Pathological	8 (34.8%)	7 (30.4%)	8 (34.8%)	23 (19.3%)		
Total	20 (16.8%)	29 (24.4%)	70 (58.8%)	119 (100.0%)		

Among the 70.6% (n = 84) of participants with a normal CTG, 83 (98.8%) neonates had an AS of 8-10 at one minute. An equivocal CTG was identified in 12 (10.1%) participants; within this group, nine (75.0%) neonates had an AS of 8-10 and three (25.0%) had a score of 4-7. Pathological CTG findings were present in 23 (19.3%) participants; in this subgroup, 10 (43.5%) neonates had an AS of 8-10, seven (30.4%) had a score of 4-7, and six (26.1%) had a score of 0-3 (Table 3). Fisher’s exact test indicated a statistically significant association between CTG analysis and the AS at one minute (p < 0.001).

**Table 3:** Relationship between CTG analysis and Apgar score in the first minute after birth *p-value obtained using Fisher’s exact test, with p < 0.05 considered significant. CTG, cardiotocography

CTG	Apgar score in the first minute			Total, n (%)	p-value*
	0-3, n (%)	4-7, n (%)	8-10, n (%)		
Normal	0 (0.0%)	1 (1.2%)	83 (98.8%)	84 (70.6%)	<0.001
Equivocal	0 (0.0%)	3 (25.0%)	9 (75.0%)	12 (10.1%)	
Pathological	6 (26.1%)	7 (30.4%)	10 (43.5%)	23 (19.3%)	
Total	6 (5.0%)	11 (9.2%)	102 (85.7%)	119 (100.0%)	

A normal BPP was predominantly associated with a normal UA pH. An equivocal BPP was most frequently associated with pre-acidosis. All pathological BPP results occurred in the acidosis group; however, acidosis was also present among normal and equivocal BPP categories (Table 4). Fisher’s exact test showed a statistically significant association between the BPP and UA pH (p < 0.001).

**Table 4:** Relationship between BPP and UA blood pH *p-value obtained using Fisher’s exact test, with p < 0.05 considered significant. UA, umbilical artery; BPP, biophysical profile

BPP	UA pH			Total, n (%)	p-value*
	Acidosis, n (%)	Pre-acidosis, n (%)	Normal, n (%)		
Normal	9 (9.8%)	20 (21.7%)	63 (68.5%)	92 (77.3%)	<0.001
Equivocal	6 (27.3%)	9 (40.9%)	7 (31.8%)	22 (18.5%)	
Pathological	5 (100.0%)	0 (0.0%)	0 (0.0%)	5 (4.2%)	
Total	20 (16.8%)	29 (24.4%)	70 (58.8%)	119 (100.0%)	

Among the 92 (77.3%) participants with a normal BPP, 91 (98.9%) neonates had an AS of 8-10 at one minute and one (1.1%) had a score of 4-7. Within the subgroup of 22 (18.5%) participants with an equivocal BPP, 11 (50.0%) neonates had an AS of 8-10, nine (40.9%) had a score of 4-7, and two (9.1%) had a score of 0-3. A pathological BPP was observed in five (4.2%) cases; in this group, one (20.0%) neonate had an AS of 4-7 and four (80.0%) had a score of 0-3 (Table 5). Fisher’s exact test indicated a statistically significant association between BPP category and the AS at one minute (p < 0.001).

**Table 5:** Relationship between BPP and Apgar score in the first minute after birth *p-value obtained using Fisher’s exact test, with p < 0.05 considered significant. BPP, biophysical profile

BPP	Apgar score in the first minute			Total, n (%)	p-value*
	0-3, n (%)	4-7, n (%)	8-10, n (%)		
Normal	0 (0.0%)	1 (1.1%)	91 (98.9%)	92 (77.3%)	<0.001
Equivocal	2 (9.1%)	9 (40.9%)	11 (50.0%)	22 (18.5%)	
Pathological	4 (80.0%)	1 (20.0%)	0 (0.0%)	5 (4.2%)	
Total	6 (5.0%)	11 (9.2%)	102 (85.7%)	119 (100.0%)	

For detecting fetal acidosis, CTG demonstrated a sensitivity of 60.0%, a specificity of 76.77%, a PPV of 34.29%, an NPV of 90.48%, and a reliability of 73.95% (Table 6). For detecting fetal acidosis, the BPP demonstrated a sensitivity of 55.0%, a specificity of 83.83%, a PPV of 40.74%, an NPV of 90.21%, and a reliability of 78.99%. For predicting a low AS at one minute, CTG showed a sensitivity of 94.12%, a specificity of 81.37%, a PPV of 45.71%, and an NPV of 98.8%, with a reliability of 83.19% (Table 7). For predicting a low AS at one minute, the BPP showed a sensitivity of 94.12%, a specificity of 89.22%, a PPV of 59.26%, and an NPV of 98.91%, with a reliability of 89.92%.

**Table 6:** Values of individual diagnostic methods (CTG and BPP) in the diagnosis of fetal acidosis BPP, biophysical profile; CTG, cardiotocography; SE, sensitivity; SP, specificity; PPV, positive predictive value; NPV, negative predictive value; FP, false positive; FN, false negative

Diagnostic method	SE	SP	PPV	NPV	Accuracy	FP	FN
CTG	60%	76.77%	34.29%	90.48%	73.95%	19.33%	6.72%
BPP	55%	83.83%	40.74%	90.21%	78.99%	13.44%	7.56%

**Table 7:** Values of individual diagnostic methods (CTG and BPP) in the prediction of a low Apgar score (0 to 7) in the first minute after birth BPP, biophysical profile; CTG, cardiotocography; SE, sensitivity; SP, specificity; PPV, positive predictive value; NPV, negative predictive value; FP, false positive; FN, false negative

Diagnostic method	SE	SP	PPV	NPV	Accuracy	FP	FN
CTG	94.12%	81.37%	45.71%	98.81%	83.19%	15.97%	0.84%
BPP	94.12%	89.22%	59.26%	98.91%	89.92%	9.24%	0.84%

Of the 119 participants, 29 (27.4%) newborns stayed in the intensive care unit for more than 24 hours after delivery and 20 (16.8%) were in acidosis-acidemia. Of these 20 neonates, nine (45.0%) had perinatal asphyxia and 11 (55.0%) did not have asphyxia. Of the six newborns who had a one-minute AS of 0-3, all six (100%) had asphyxia; among the 11 newborns with an AS of 4-7 at birth, six had perinatal asphyxia (54.5%).

Overall, two children died within the first seven days after delivery and one child died on the 20th postpartum day. The two children who died within seven days were in acidosis at birth, had a low AS at birth, had pathological CTG findings, and had pathological arterial and venous flows. Based on these data, we calculated perinatal mortality and morbidity (1.68% and 24.37%, respectively).

## Discussion

Our findings indicate that CTG and the BPP are statistically significant tools for assessing fetal oxygenation, with similar predictive value for fetal acidosis and a low AS. Both tests showed moderate sensitivity for fetal acidosis and high sensitivity for a low AS, supporting their clinical utility.

CTG produced somewhat higher FP rates for fetal acidosis and a low AS than the BPP, consistent with published data [14]. Both tests had low FN rates, particularly for predicting a low AS, indicating high diagnostic efficacy for identifying fetal acidosis and low AS in this cohort.

Like other diagnostic methods, CTG can yield FP and FN results. An FP CTG occurs when the tracing appears pathological, but a non-depressed, healthy neonate with a normal acid-base status is born. An FN CTG occurs when the tracing appears normal, but an asphyxiated neonate is delivered.

Published studies report variable performance metrics. Škrablin [14] analyzed intrapartum CTG in neonates with verified hypoxic-ischemic encephalopathy and found FN results in 29.0% and FP results in 17.5%. Spencer [15] reported 52.8% FN and 38.9% FP results, while Nelson and Ellenberg [16] reported 73.1% FN and 9.3% FP results.

We observed a significant correlation between pathological CTG and fetal acidosis. However, literature suggests a weaker association between CTG changes and an AS < 7 and a stronger association between a pathological CTG and fetal acidosis, which does not fully align with our results [17]. Kuvačić [18] reported UA acidosis in 44.4% of cases with pathological CTG; in our study, acidosis followed a pathological CTG in 34.8% of participants.

Additional evidence supports integrating biophysical testing. Baschat [19] found that an abnormal BPP in fetal growth restriction more accurately predicts intrapartum acidemia and, together with pathological CTG (decelerations), serves as a robust indicator at any gestational age. Attini et al. [20] reported that 49% of neonates with acidosis had a normal CTG, indicating limited CTG specificity for identifying acidosis at birth and in neonates at risk for neurologic sequelae. Baschat et al. [21] also reported that, depending on cohort, 30% and 70% of fetuses with a non-reactive CTG require a BPP to confirm fetal condition, and that an abnormal BPP score in 8% to 27% of cases is an indication for delivery. Sapoval et al. [22] concluded that the BPP is a non-invasive, easily applicable, and highly specific predictor of fetal condition in high-risk pregnancies.

Antenatal fetal surveillance relies on tests that detect abnormal changes in fetal behavior and physiology. The goal is to identify at-risk fetuses with biophysical tests to enable timely intervention and reduce adverse perinatal outcomes.

Limitations

Our study had several important limitations. This single-center observational study of 119 hospitalized pregnancies may not generalize to lower-risk or outpatient populations; inclusion criteria (gestational age ≥ 27 weeks, singleton gestation, no congenital malformations) further limit applicability to earlier gestations, multiple pregnancies, and fetuses with structural anomalies. The interpretation of CTG and BPP is partly subjective; we did not assess interobserver or intraobserver agreement, nor did we ensure reader blinding. The use of a specific device and the Kuvačić-Dražančić index may reduce comparability with centers using other systems. Outcome assessment relied on UA pH categories and the one-minute AS, both influenced by perinatal management; pre-analytical factors affecting blood gas results were not systematically recorded, and lactate was not a primary classifier. We analyzed only the final same-day CTG/BPP; the interval to birth, intrapartum events, and interventions were not modeled, and predictive values (being prevalence-dependent) may not transport to other settings. We did not perform multivariable adjustment, discrimination or calibration analyses, decision-analytic evaluation, subgroup analyses, or test the incremental value of combining CTG and BPP. Follow-up was limited to the early postpartum period, without hypoxic-ischemic encephalopathy staging, neuroimaging, or longer-term neurodevelopmental outcomes.

## Conclusions

This study evaluated the diagnostic performance of CTG and the BPP for assessing fetal oxygenation. Both modalities correlated with UA pH and AS and demonstrated useful diagnostic and predictive performance for fetal hypoxia, acidosis, and asphyxia. Adverse perinatal outcomes clustered with pathological findings on either test and with biochemical evidence of acidosis. These results support the integrated use and careful interpretation of CTG and BPP as complementary tools for antenatal and intrapartum surveillance to identify at-risk fetuses and to guide timely decisions about the mode and timing of delivery.
